# Hepatic Cellular Senescence Is Attenuated by Filbertone via Modulation of the p53-p21 Pathway in AML12 Hepatocytes

**DOI:** 10.3390/nu18142278

**Published:** 2026-07-11

**Authors:** Sujung Park, Byungyong Ahn

**Affiliations:** 1 Department of Food Science and Nutrition, University of Ulsan, 93 Daehak-ro, Nam-gu, Ulsan 44610, Republic of Korea; 2 Basic-Clinical Convergence Research Institute, University of Ulsan, Ulsan 44610, Republic of Korea

**Keywords:** aging, filbertone, senescence, p53, p21, SASP

## Abstract

**Background/Objectives**: Cellular senescence refers to a state where the phenotype of cells changes, and the cell cycle comes to a halt. Cellular aging occurs due to various causes and is implicated in the development of various age-related diseases. While the component filbertone in hazelnuts is known for its anti-obesity, anti-neurodegenerative, and anti-inflammatory effects, its potential anti-aging effects have not been elucidated. This study investigated whether filbertone modulates cellular senescence via the tumor suppressor protein p53 and its downstream target p21 in AML12 hepatocytes. **Methods**: AML12 hepatocytes were treated with doxorubicin or hydrogen peroxide, with or without filbertone (25–100 μM), in both an acute stress-induction model and an established senescent-cell model. p53 and p21 expression, senescence-associated β-galactosidase (SA-β-gal) staining, and the expression of senescence-associated secretory phenotype (SASP)-related cytokines (Il-1β, Il-6, Tnf-α) were assessed. **Results**: The results demonstrated that filbertone dose-dependently reduced the increased p53 and p21, a downstream gene of p53, induced by doxorubicin and hydrogen peroxide. Furthermore, the number of hepatic senescent cells with senescence-associated β-galactosidase staining was significantly decreased in the presence of filbertone. Filbertone treatment also significantly suppressed the expression of senescence-associated secretory phenotype (SASP)-related pro-inflammatory cytokines, including Il-1β, Il-6, and Tnf-α, in both stress-induced and established senescent hepatocytes. **Conclusions**: These findings indicate that filbertone modulates senescence-associated p53–p21 signaling and the accompanying inflammatory secretory phenotype in an in vitro model of AML12 hepatocytes, suggesting that filbertone may be a geroprotective compound that attenuates cellular senescence.

## 1. Introduction

The liver, a vital organ with critical functions such as metabolism, detoxification, and synthesis of essential proteins, undergoes substantial changes as the body ages [1,2]. Although the liver displays remarkable resilience and regenerative capabilities, the process of aging results in a gradual decline in its physiological capacity and an increased susceptibility to liver-related diseases [3,4]. It is of paramount importance to gain an understanding of the changes that occur in the liver as a result of the aging process. This is not only due to the direct impact that these changes have on hepatic function, but also because of the liver’s role in maintaining overall health and well-being [5].

One of the most salient cellular changes in the aging liver is the accumulation of senescent cells. A comprehensive understanding of senescence-related liver changes is imperative, as it elucidates the mechanisms underlying age-related hepatic dysfunction and informs the development of strategies to manage liver health in the elderly population. Cellular senescence, defined as a state of irreversible growth arrest that cells enter in response to damage or stress, is a hallmark of the aging process [6,7]. Senescent cells within the liver exhibit a decline in their functional capabilities, accompanied by the secretion of pro-inflammatory cytokines and growth factors, a phenomenon known as the senescence-associated secretory phenotype (SASP) [8,9]. SASP factors have been shown to induce chronic inflammation within the liver, thereby increasing susceptibility to various liver diseases and contributing to a state of systemic inflammation termed inflammaging [10]. This inflammatory state has been associated with an increase in liver tissue damage and a concomitant decrease in regenerative capacity [11]. These cellular and molecular changes are subject to rigorous regulation by various signaling pathways. A notable example is the p53–p21 pathway, which plays a crucial role in preventing uncontrolled cell proliferation and DNA damage. The tumor suppressor protein p53 is a key mediator of these responses, and its activation occurs in response to DNA damage or oxidative stress, which are prevalent in aged cells. Upon activation, p53 can initiate the expression of p21, a cyclin-dependent kinase inhibitor. This results in cell cycle arrest and enables cells to repair DNA damage or enter a senescent state [12,13,14].

In addition to its impact on cellular proliferation, the p53–p21 pathway contributes to the metabolic alterations observed in the aging liver. Moreover, the p53–p21 pathway impacts the hepatocellular regenerative capacity, which undergoes a decline with advanced age. The liver’s capacity for regeneration following injury has been extensively documented. However, with advancing age, this regenerative potential is impaired due to heightened levels of p53 and p21. Elevated levels of p21 have been shown to inhibit cell cycle progression, resulting in a decrease in the rate of hepatocyte proliferation necessary for tissue repair [15].

Oxidative stress and DNA damage are recognized as central upstream triggers of hepatocyte senescence: excessive reactive oxygen species (ROS) generation and unresolved DNA lesions activate the DNA damage response, converging on p53 stabilization and downstream p21 induction, while sustained ROS accumulation can also engage the p16INK4a-Rb axis, a parallel senescence-associated cell-cycle-arrest pathway. The resulting senescent hepatocytes acquire the SASP, secreting pro-inflammatory cytokines such as IL-6, IL-1β, and TNF-α that reinforce the arrest in an autocrine manner and propagate senescence and inflammation to neighboring cells in a paracrine fashion, thereby amplifying hepatic inflammaging [8,9,10,11]. Counterbalancing this oxidative burden, the nuclear factor erythroid 2-related factor 2 (Nrf2) pathway serves as a master regulator of antioxidant and cytoprotective gene expression, and its decline has been linked to accelerated cellular senescence. Notably, filbertone has previously been shown to activate ROS-responsive PERK-TFEB autophagy-lysosomal signaling and Nrf2-dependent antioxidant gene expression in neuronal cells [16,17], raising the possibility that similar redox-modulatory mechanisms could underlie its effects in other tissues, including the liver. Although hazelnuts and hazelnut-derived compounds are commonly consumed as part of diets associated with favorable metabolic and hepatic outcomes, whether filbertone [(E)-5-methyl-2-hepten-4-one], the principal flavor-active constituent of hazelnuts and a generally-recognized-as-safe (GRAS) food-derived compound [18], can directly modulate hepatocyte senescence has not previously been examined. Given that the liver is both a primary site of xenobiotic and dietary compound metabolism and a tissue highly susceptible to senescence-driven functional decline with age, this represents an important and previously unaddressed knowledge gap.

Filbertone, a naturally occurring compound in hazelnuts, has been the focus of research due to its potential health benefits, though further study is necessary to fully elucidate its physiological roles. Recent studies have indicated its ability to reduce blood glucose levels, decrease body weight and visceral fat, prevent obesity-induced hypothalamic inflammation, and ameliorate neurodegenerative diseases [16,19,20]. Additionally, filbertone shows promise in mitigating neurodegenerative diseases, and its role in regulating muscle lipid metabolism underscores its contribution to maintaining energy balance and overall metabolic health [17,21,22]. However, despite the encouraging findings, there is currently no conclusive evidence directly linking filbertone to hepatic aging or its associated physiological mechanisms. Therefore, the objective of the present study was to determine whether filbertone modulates senescence-associated p53–p21 signaling and the accompanying pro-inflammatory SASP response in doxorubicin- and H_2_O_2_-induced, as well as in established, senescence models of AML12 hepatocytes.

## 2. Materials and Methods

### 2.1. Reagents

Fetal bovine serum (FBS), Penicillin-streptomycin, Dulbecco’s modified Eagle medium (DMEM), DPBS, PBS, and Trypsin-EDTA were purchased from Welgene (Gyungsan, Republic of Korea). Filbertone (5-Methyl-2-hepten-4-one, 98%), doxorubicin, hydrogen peroxide (H_2_O_2_), ammonium persulfate, chloroform, TWEEN 20, phosphatase inhibitor, thiazolyl blue tetrazolium bromide (MTT), dimethyl sulfoxide (DMSO), dithiothreitol (DTT), ethanol, methanol, and skim milk powder were purchased from Sigma-Aldrich (St. Louis, MO, USA). The cOmplete^TM^ protease inhibitor cocktail was purchased from Roche (Indianapolis, IN, USA). Trizol, RIPA buffer, and Micro BCA Protein Assay Kit were obtained from Thermo Fisher Scientific (Waltham, MA, USA). Sodium dodecyl sulfate (SDS) was purchased from Bio-rad (Hercules, CA, USA). Senescence β-Galactosidase Staining kit were obtained from Cell signaling (Danvers, MA, USA). Westar Antares ECL substrate was purchased Cyanagen (Bologna, Italy). TB Green^®^ Premix Ex Taq II was purchased by TaKaRa Bio Inc. (Foster, CA, USA). MMLV RT buffer (10X), dNTP (10 mM), Rnase inhibitor, Random primer, MMLV RT Enzyme were purchased from Promega (Madison, WI, USA). Filbertone and doxorubicin stock solutions were prepared in DMSO; the final DMSO concentration in the culture medium was kept at or below 0.1% (*v*/*v*) across all experimental groups, including vehicle-treated controls.

### 2.2. Cell Culture

Alpha mouse liver 12 (AML-12) cells were supplied by ATCC (CRL-2254; Manassas, VA, USA) and were cultured in Dulbecco’s modified Eagle medium (DMEM) with 10% fetal bovine serum (FBS) and 1% Penicillin-streptomycin, which were maintained in a humidified atmosphere containing 5% CO_2_ at 37 °C.

### 2.3. Induction of an Acute Senescence-Associated Stress Response

**Figure 1:**
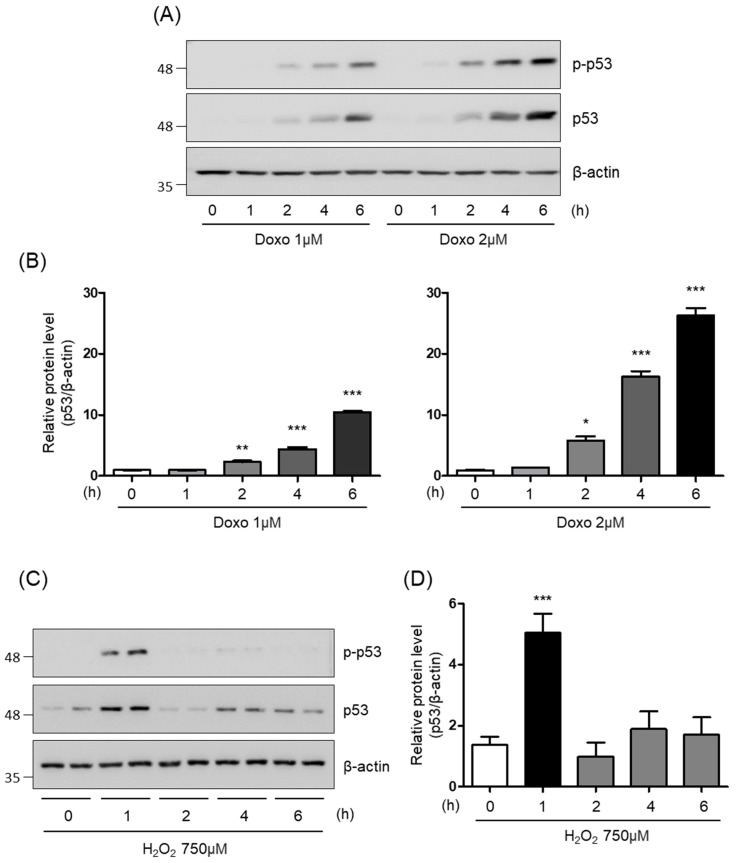
**Cellular senescence is derived from doxorubicin or hydrogen peroxide in AML12 hepatocytes.** (**A**) Doxorubicin (Dox; 1 or 2 μM) or (**C**) H_2_O_2_ (750 μM) was treated in AML12 hepatocytes for the indicated times. The protein expression of p53, p-p53, and β-actin were determined by western blot analysis. Protein levels were normalized to β-actin levels in each sample (**B**,**D**). The graph shows the quantification of Western blot data (*n* = 4 per group). All results are presented as mean ± SD. * *p* < 0.05, ** *p* < 0.01, *** *p* < 0.005 compared to 0 h by one-way ANOVA with Tukey multiple comparison post hoc test.

**Figure 2:**
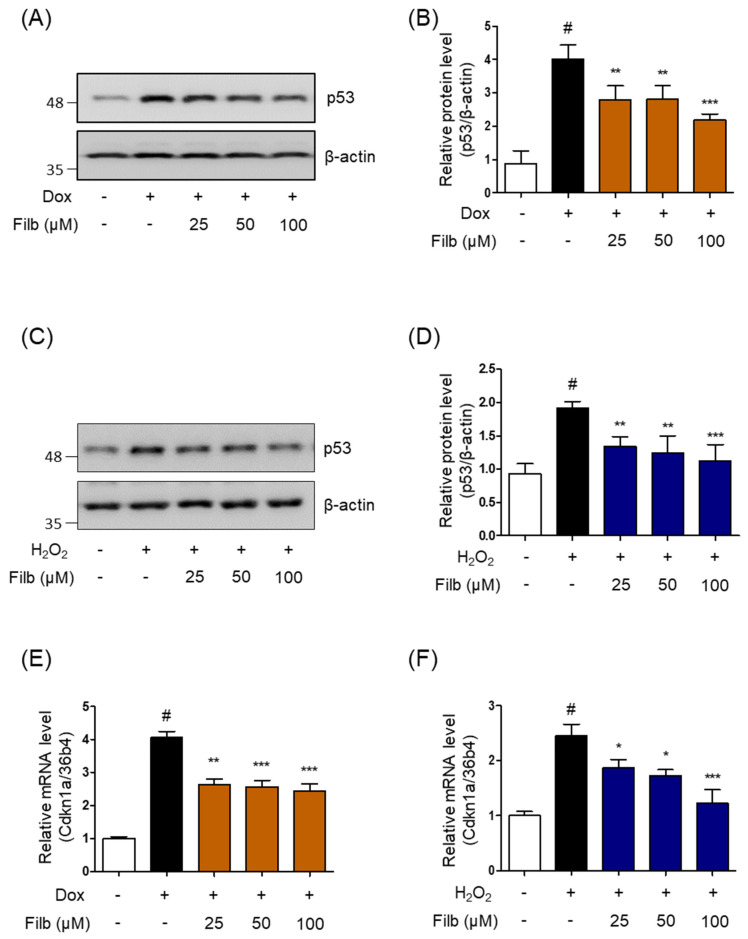
**Filbertone regulates cellular senescence in AML12 hepatocytes.** AML12 hepatocytes were treated with doxorubicin 2μM (6 h) or H_2_O_2_ 750 μM (1 h) in the presence of filbertone (Filb; 0, 25, 50, 100 μM) for 24 h. The levels of p53 protein were determined by western blot analysis (**A**,**C**) and were normalized to the β-actin in each sample ((**B**,**D**); *n* = 4 per group). The gene expression of Cdkn1a (p21) was measured by qRT-PCR and was normalized to the 36b4 expression in each sample ((**E**,**F**); *n* = 4 per group). Results are presented as mean ± SD. # *p* < 0.001 compared to Veh; * *p* < 0.05, ** *p* < 0.01, *** *p* < 0.005 compared to Dox (only) or H_2_O_2_ (only) by one-way ANOVA with Tukey multiple comparison post hoc test.

To induce an acute senescence-associated stress response, AML12 hepatocytes were treated with doxorubicin (1 or 2 μM) or H_2_O_2_ (750 μM) for the indicated durations, as detailed in the corresponding figure legends. For the experiments shown in Figure 1 and Figure 2 and 5A–C, cells were treated with doxorubicin (2 μM) for 6 h or H_2_O_2_ (750 μM) for 1 h, in the presence or absence of filbertone (25–100 μM) as indicated. These treatment durations were selected based on the time-course analyses shown in Figure 1 and Supplemental Figures S1–S3, which identified these time points as producing robust and reproducible induction of p53/p-p53.

### 2.4. Generation of Senescent Cells from the Mouse Hepatic Cell Line AML12

As previously outlined in the literature, the generation of senescent cells was induced through the implementation of oxidative stress [23]. Briefly, AML12 cells for oxidative stress induction were seeded at a density of 1.0 × 10^6^ cells/well in a 100 mm cell culture dish and incubated for 24 h. After confirming cellular growth at approximately 50–60%, oxidative stress was induced by treating the cells with 1.0 mM H_2_O_2_ for 1 h. The initiation of the 1 h treatment with 1.0 mM H_2_O_2_ was designated as day 1, representing the induction of oxidative stress. Following the 1 h treatment, the cells underwent a recovery period of 23 h. Subsequently, from day 2 to day 6, cells were treated daily with 750 μM H_2_O_2_ for 1 h at the same time each day, followed by a 23 h recovery period. The experimental endpoint, marked as day 7, was chosen as the point of cellular senescence. At this juncture, cells were cultured for subsequent experimental procedures.

### 2.5. Cell Viability Assay

Cell viability was assessed using a 3-(4,5-dimethyl-2-thiazolyl)- 2,5-diphenyl-2H-tetrazolium bromide (MTT) assay (Thermofisher; Thermo Fisher Scientific, Waltham, MA, USA). For the assay, 0.4 × 10^5^ cells/well of AML12 cells were seeded on 48-well flat plates for 24 h and then pretreated with various concentrations of filbertone (25, 50, 100 µM) for 48 h. After the treatment, 250 µL of MTT solution was added to each well for 1 h of incubation. Then, the supernatant was removed and the colored crystals of formazan were dissolved with 200 µL of dissolving solution (DMSO). The optical density (OD) was measured using a microplate reader ( Biotek Synergy HTX; Agilent, Winooski, VT, USA) at 570 nm. The filbertone concentration range (25–100 μM) was selected based on our previous studies demonstrating anti-senescent efficacy without cytotoxicity at comparable doses in murine C_2_C_12_ myotubes [22], and its non-cytotoxicity was independently confirmed in AML12 hepatocytes in the present study prior to its use in subsequent senescence-modulation experiments (Supplemental Figure S2).

### 2.6. Senescence-Associated β-Galactosidase (SA-β-Gal) Staining

The SA-β-gal staining procedure was carried out on normal AML12 cells and on senescent cells derived from AML12 hepatocytes. The staining was performed using a Senescence β-Galactosidase Staining Kit (Cat. No. #9860; Cell Signaling Technology, Inc., Danvers, MA, USA), following the manufacturer’s instructions. Bright-field images of SA-β-gal-stained cells were captured using an Olympus CK2 inverted microscope (Olympus, Tokyo, Japan) equipped with an Infinity1 camera (Lumenera Corporation, Ottawa, ON, Canada). The density of SA-β-gal-positive cells for each group was subsequently quantified using the ImageJ software(Ver. 1.54q) provided by the National Institutes of Health (NIH). To calculate the number of SA-β-gal-positive cells, the total number of cells and the number of SA-β-gal-positive cells were counted, and the ratio of SA-β-gal-positive cells to total cells was subsequently calculated. This quantification approach follows previously established protocols for SA-β-gal-based senescence assessment [24]. p53/p21 induction, together with SA-β-gal positivity, were used as core, widely accepted markers to define the senescent phenotype in this study [25].

### 2.7. Protein Sample Preparation and Western Blotting

Cells were lysed by radioimmunoprecipitation assay (RIPA) buffer (Thermo Fisher Scientific) containing phosphatase inhibitor (Phostop; Roche) and protease inhibitor cocktails (cOmplete; Roche). Protein concentration was determined by using Micro BCA Protein Assay Kit (Thermo Fisher Scientific). 20–30 µg of protein was subjected to 8–10% sodium dodecyl sulfate-polyacrylamide gel (SDS-PAGE) and transferred to a nitrocellulose membrane. Membranes were blocked with 5% (*w*/*v*) skim milk solution for 1 h at room temperature and incubated with primary antibodies overnight at 4 °C as follows: anti-p53 and anti-phospho-p53 were obtained from Cell Signaling Technology (Danvers, MA, USA). Anti-β-actin was purchased from Sigma-Aldrich. After washing with Tris-buffered saline and Tween 20 (0.1% TBS-T) buffer 3 times for 15 min, membranes were incubated for 1 h in the appropriate secondary antibody added in antibody diluent at room temperature. Membranes were washed again 3 times with 0.1% TBS-T for 15 min. The quantification of protein levels was measured by Westar Antares (Cyanagen). Protein detection and quantification were conducted using Fusion Solo S (Vilber, Marne-la-Vallée, France). The intensity of bands was normalized to that of β-actin.

### 2.8. RNA Isolation and Quantitative RT-PCR (qRT-PCR)

Total RNAs were extracted from cells using TRIzol Lysis reagent (Invitrogen, Carlsbad, CA, USA) according to the manufacturer’s instructions. To generate cDNAs, 2.0 µg of total RNA was used in a reaction with M-MLV reverse transcriptase (Promega). Polymerase chain reaction (PCR) reactions were subsequently conducted using TB Green Premix Ex Taq II on a Thermal Cycler Dice Real-Time PCR System (TaKaRa Bio Inc., Otsu, Shiga, Japan) with specific primers for each gene. The analysis of all qRT-PCR data was conducted using TP800 software (TaKaRa Bio Inc.). The primer sets utilized are detailed in Supplemental Table S1. The expression of target mRNAs was then normalized by that of *Rplp0* (36B4) as the standard.

### 2.9. Statistical Analysis

Statistical comparisons among experimental groups were conducted using one-way analysis of variance (ANOVA) followed by Tukey’s multiple comparisons post hoc test, as specified in each figure legend. Sample sizes (*n* = 3–5 biological replicates per group) are indicated in the corresponding figure legend for each experiment. Significance levels are denoted as * *p* < 0.05, ** *p* < 0.01, *** *p* < 0.005 for comparisons specified within each panel; the symbol # denotes comparison to the vehicle-treated or normal control group, with the exact threshold (ranging from *p* < 0.001 to *p* < 0.01 depending on the experiment) specified in the corresponding figure legend. Non-significant results were indicated as NS (not significant). Statistical analyses were performed by using GraphPad Prism 8.0 software (San Diego, CA, USA).

## 3. Results

### 3.1. Establishment of a Model of Cellular Senescence in AML12 Hepatocytes

A study was conducted with the objective of examining the cellular senescence signaling pathway in the liver. To investigate this pathway, a senescence-induced cell model was generated through the administration of doxorubicin or hydrogen peroxide. The protein levels of p53 (tumor protein p53), a well-known senescence marker, were monitored under various conditions. In addition, the levels of phosphorylated p53 (p-p53), the activated form of p53, were assessed under different conditions. We found that both p53 and p-p53 are induced by doxorubicin in a dose-dependent manner, but not in a time-dependent manner (Supplemental Figure S1). Moreover, the results showed that hydrogen peroxide time-dependently enhances both p53 and p-p53 (Supplemental Figure S3). The results of this study revealed a rapid and significant increase in p53 protein levels within 6 h of incubating the cells with doxorubicin 2 μM (Figure 1A,B) or 1 h in the presence of hydrogen peroxide 750 μM (Figure 1C,D). Therefore, these findings suggest a potential involvement of p53 protein levels in the senescence process of hepatocyte cells.

### 3.2. Effect of Filbertone in Senescence-Induced AML12 Cells Treated with Doxorubicin or Hydrogen Peroxide

The objective of the present study was to ascertain whether filbertone exerts a regulatory influence on the protein levels of p53 in senescence-induced AML12 hepatocytes. The results demonstrated that filbertone diminished the level of p53 protein induced by doxorubicin in a dose-dependent manner, as illustrated in Figure 2A,B. A similar dose-dependent effect was observed in the reduction of hydrogen peroxide-induced p53 protein levels by filbertone, as shown in Figure 2C,D. In the context of these findings, it is important to note that previous reports have identified p21 as one of the primary targets of p53 [26,27,28]. Therefore, the subsequent investigation focused on determining whether filbertone regulates the expression of p21 in senescence-induced AML12 hepatocytes with doxorubicin or hydrogen peroxide. The results of this investigation revealed that filbertone significantly and dose-dependently downregulates the gene expression of p21 in senescent liver cells (Figure 2E,F). Furthermore, we examined the impact of filbertone on the viability of AML12 hepatocytes, assessing its effect in a dose-dependent fashion. The results demonstrated that filbertone did not exhibit any signs of toxicity in AML12 hepatocytes (Supplemental Figure S3). Collectively, these findings suggest that the p53–p21 pathway, cellular senescence signaling, is modulated by filbertone in liver cells.

### 3.3. Effect of Filbertone in Senescent Cells Derived from AML12 Hepatocytes

**Figure 3:**
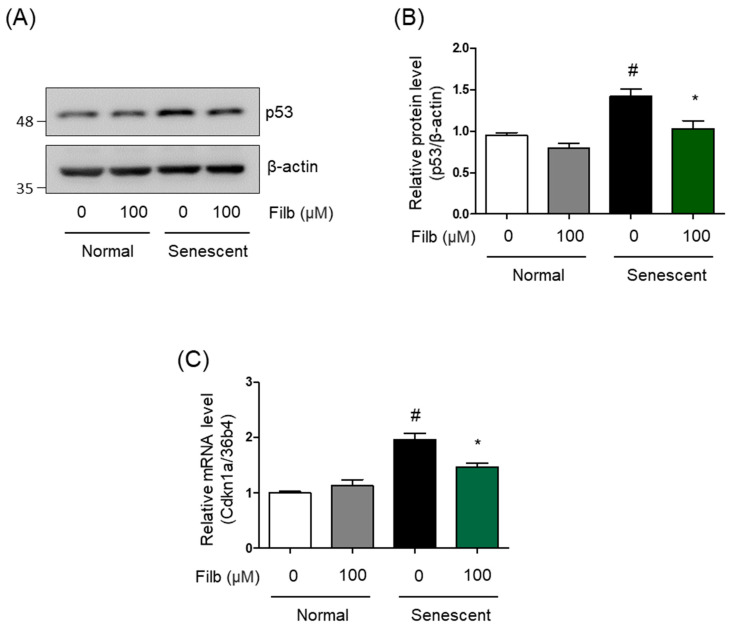
**The effect of filbertone in senescent cells derived from AML12 hepatocytes.** Normal or senescent cells were treated with filbertone for 24 h in order to assess the levels of the p53 protein ((**A**,**B**); *n* = 4 per group) and the expression of p21 (Cdkn1a) mRNA ((**C**); *n* = 4 per group). The data represent mean ± SD. # *p* < 0.01 vs. filbertone 0 µM in normal, and * *p* < 0.05 compared with filbertone 0 µM in senescent by one-way ANOVA with Tukey’s multiple-comparisons post hoc test.

Hepatic senescent cells were generated from AML12 hepatocytes (see the detailed description in the method). We next sought to assess whether the process of cellular senescence is controlled by filbertone in hepatic senescent cells. As anticipated, the protein levels of p53 were induced in the senescent cells. Then, the induction of p53 protein was diminished by filbertone in the senescent cells (Figure 3A,B). Additionally, the gene expression of p21 (Cdkn1a) was observed to be augmented in the senescent cells. The induction of p21 gene expression was repressed by filbertone (Figure 3C). These observations collectively imply that filbertone exerts a regulatory effect on the progression of hepatic cellular senescence in senescent cells.

### 3.4. The Activity of Senescence-Associated β-Galactosidase (SA-β-Gal) Is Affected by Filbertone in Hepatic Senescent Cells

**Figure 4:**
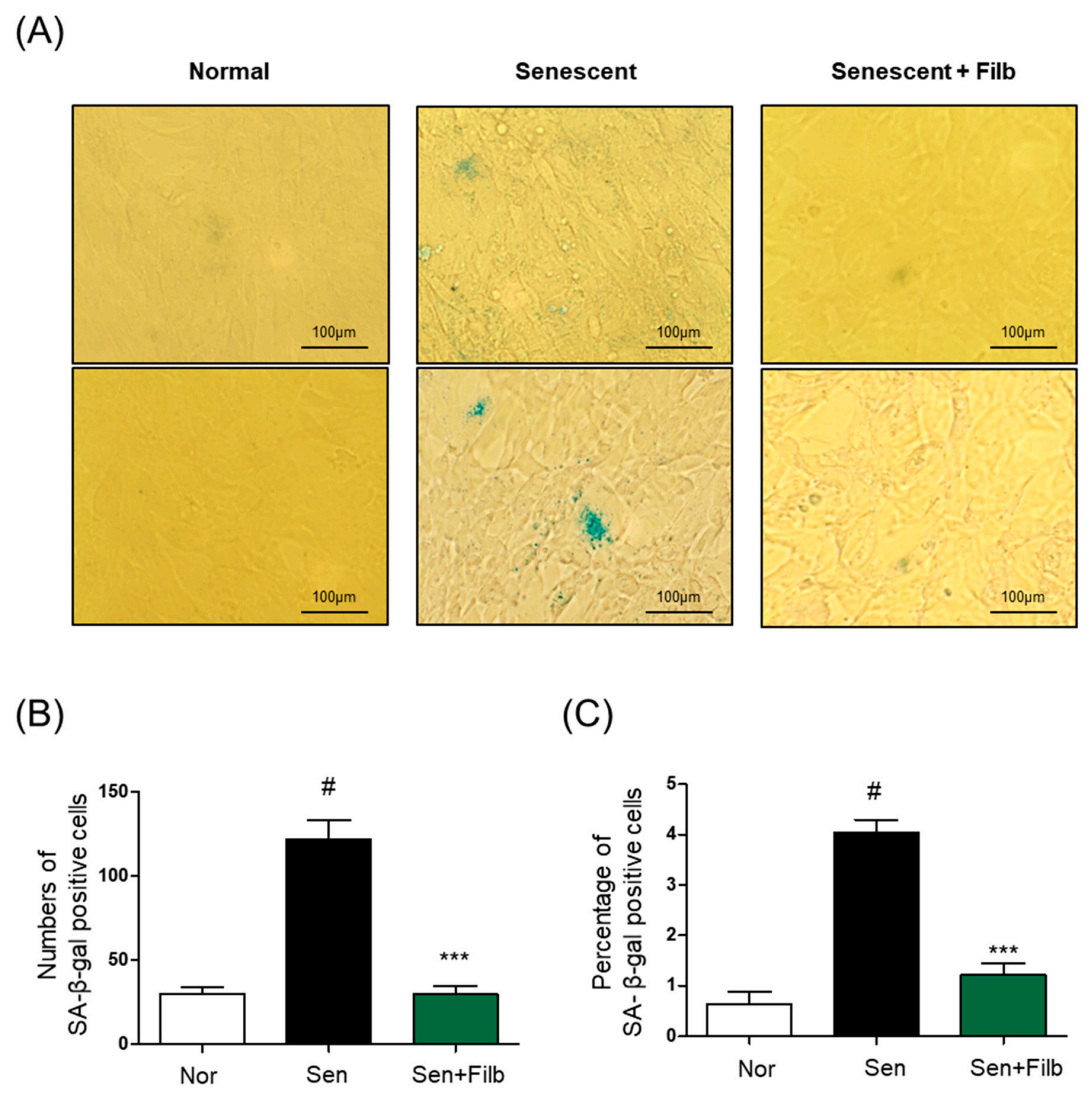
**Senescence-associated β-galactosidase staining of senescent cells derived from AML12 hepatocytes.** (**A**) Representative morphologies of senescence-associated β-galactosidase (SA-β-gal) stained (bluish-green color) in normal (Nor) AML12 cells, senescent (Sen) cells, and senescent cells with filbertone (Filb) for 24 h. Scale bar = 100 μm. SA-β-gal quantification plots based on the count of positive cells (**B**) and the percentage (**C**) of area (*n* = 4 per group). The data represent mean ± SD. # *p* < 0.005 vs. normal, and *** *p* < 0.005 vs. senescent with filbertone 100 µM by one-way ANOVA with Tukey’s multiple-comparisons post hoc test.

The assay for the identification of senescent cells, known as the senescence-associated β-galactosidase (SA-β-gal) staining assay, is considered a highly reliable technique in the field [26,29,30]. A method for evaluating the presence of cellular senescence in hepatic senescent cells was developed. This approach entailed the use of SA-β-gal staining to identify the hallmarks of cellular senescence. We aimed to investigate the role of filbertone in the regulation of the signaling pathway of cellular senescence in hepatic senescent cells. To this end, the SA-β-gal staining assay was conducted in the senescent cells under both conditions of filbertone presence and absence. The intensity of SA-β-gal staining, an indicator of senescence, was observed to decrease significantly in hepatic senescent cells upon treatment with filbertone, to levels similar to those observed in normal AML12 cells (Figure 4A). Furthermore, it was observed that both the number and the percentage of senescent cells were significantly attenuated by filbertone (Figure 4B). Hence, these results further demonstrated that filbertone modulates the senescence signaling pathway in hepatic senescent cells.

### 3.5. Filbertone Suppresses SASP-Associated Inflammatory Cytokine Expression in AML12 Hepatocytes

**Figure 5:**
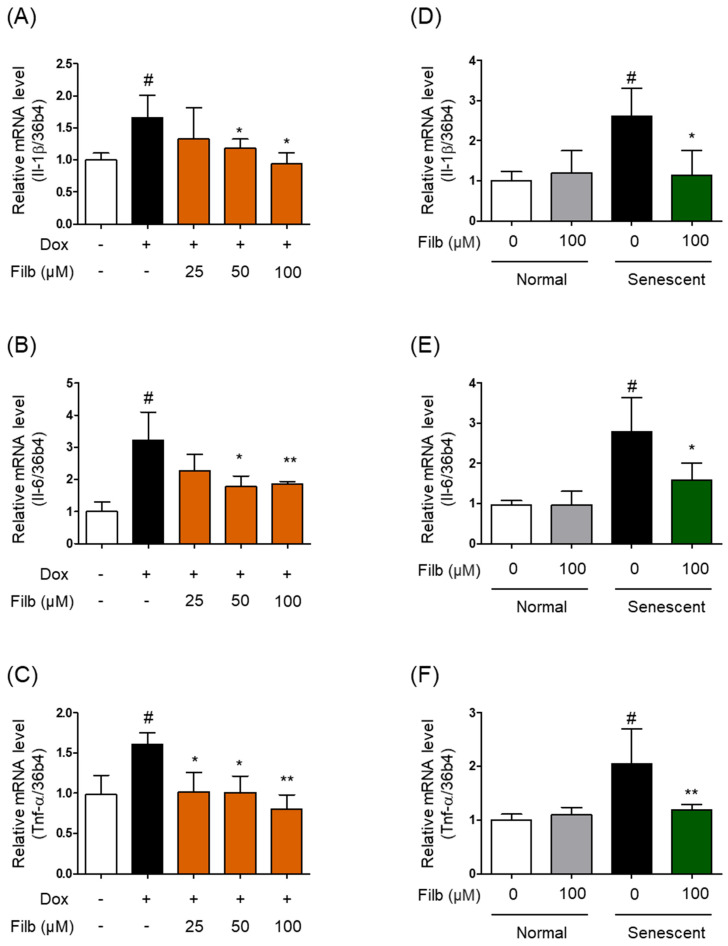
**Suppression of SASP-associated inflammatory cytokines by filbertone.** (**A**–**C**) AML12 hepatocytes were treated with doxorubicin 2μM (6 h) in the presence of filbertone (Filb; 0, 25, 50, 100 μM) for 24 h. The expression of genes (Il-1b, Il-6 and Tnf-a) was measured by qRT-PCR and was normalized to the 36b4 expression in each sample (*n* = 5 per group). Results are presented as mean ± SD. # *p* < 0.01 compared to Veh; * *p* < 0.05, ** *p* < 0.01 compared to Dox (only) by one-way ANOVA. (**D**–**F**) Normal or senescent cells were treated with filbertone for 24 h in order to assess the expression of genes including Il-1b, Il-6 and Tnf-a (*n* = 5 per group). The data represent mean ± SD. # *p* < 0.01 vs. filbertone 0 µM in normal, and * *p* < 0.05, ** *p* < 0.01 compared with filbertone 0 µM in senescent by one-way ANOVA with Tukey’s multiple-comparisons post hoc test.

Beyond cell-intrinsic senescence markers, senescent cells characteristically acquire a senescence-associated secretory phenotype (SASP), marked by the secretion of pro-inflammatory cytokines that propagate senescence and inflammation within the tissue microenvironment [8,9,10,11]. We therefore examined whether filbertone also regulates the expression of representative SASP factors, including Il-1β, Il-6, and Tnf-α. In AML12 hepatocytes treated with doxorubicin (2 μM, 6 h), the mRNA expression levels of Il-1β, Il-6, and Tnf-α were significantly elevated relative to vehicle-treated controls, and this induction was significantly and dose-dependently attenuated by co-treatment with filbertone (Figure 5A–C). Consistent with these findings, the expression levels of Il-1β, Il-6, and Tnf-α were also significantly increased in hepatic senescent cells compared with normal AML12 cells, and filbertone treatment (100 μM) significantly suppressed this senescence-associated induction (Figure 5D–F). Taken together, these results indicate that filbertone attenuates not only the core p53–p21 senescence signaling axis but also the associated pro-inflammatory SASP response in hepatocytes, suggesting a broader anti-inflammatory dimension to its anti-senescent activity.

## 4. Discussion

The aging of the liver is a complex process that is influenced by a number of factors, including cellular, molecular, and structural changes. The liver undergoes structural and histologic changes with age. Studies have shown that liver volume and weight tend to decrease progressively after the age of 50. This phenomenon is attributed to the reduced cell size and number as hepatocytes undergo apoptosis and are not adequately replaced [1,31]. These changes ultimately lead to a reduction in the liver’s functional capacity, a decline in its regenerative abilities, and an increased susceptibility to the development of various pathologies, such as fibrosis, steatosis, and cancer [4,32,33]. In the present study, it was demonstrated that filbertone treatment significantly attenuated hepatic cellular senescence, thereby confirming the initial hypothesis. However, cellular senescence—the cell-autonomous growth-arrest state examined here—is only one contributor to, rather than a synonym for, organismal liver aging. Therefore, the present findings suggest that filbertone modulates senescence-associated signaling pathways, rather than providing direct evidence of anti-aging or life-extending effects at the whole-organism level.

As the liver undergoes the process of aging, it exhibits a decline in efficiency in lipid and glucose metabolism, which, in turn, leads to an increased risk of metabolic disorders, including insulin resistance and nonalcoholic fatty liver disease (NAFLD) [34,35]. A primary regulatory pathway involved in cellular senescence and aging in the liver is the p53–p21 axis. This pathway plays a pivotal role in maintaining cellular integrity, orchestrating processes such as cell cycle arrest, DNA repair, and apoptosis [36,37]. The activation of p53 in response to metabolic stress has been demonstrated to regulate various genes involved in metabolism, thereby further contributing to hepatic insulin resistance and lipid dysregulation [38]. A comprehensive understanding of the role of the p53–p21 pathway in liver aging is essential for elucidating the molecular mechanisms underlying hepatic senescence [39]. It underscores the delicate balance that the pathway maintains between protecting against cellular damage and contributing to functional decline. Consequently, research directed towards the modulation of the p53–p21 pathway may yield therapeutic benefits for addressing age-related hepatic dysfunction and augmenting hepatic resilience in elderly individuals [26].

In the current study, we investigated whether filbertone could possibly influence the regulation of the liver aging process in AML12 hepatocyte cells exposed to senescence induction by the treatment with either doxorubicin or hydrogen peroxide. p53 plays a central role in the induction and regulation of cellular senescence through a variety of mechanisms, including DNA damage and oxidative stress. We have identified that p53 is significantly increased in AML12 hepatocytes by administration of doxorubicin for 6 h or hydrogen peroxide for 1 h. In senescent liver cells, p53 is also significantly upregulated. Filbertone treatment led to a downregulation of p53 levels in senescence-induced AML12 hepatocytes, as well as in the senescent cells. Levels of p21, a primary target of p53, were also successfully repressed by filbertone treatment. Furthermore, our results showed that the activity of senescence-associated β-galactosidase (SA-β-gal), a well-established method for the identification of aging cells, is suppressed by filbertone. Overall, these results suggest that filbertone has an important effect on the aging process of the liver. These conclusions, however, rest on a core but limited marker set (p53, p21, and SA-β-gal activity); complementary endpoints such as p16INK4a expression, γH2AX foci, and cell-cycle profiling were not examined in this study [25], and their inclusion would allow a more complete characterization of the senescent phenotype and of filbertone’s effects on it.

Although the present study establishes a clear association between filbertone treatment and reduced p53–p21 signaling, the precise upstream mechanism by which filbertone achieves this effect remains to be defined. Because both doxorubicin and H_2_O_2_ induce senescence primarily through ROS generation and consequent DNA damage-response activation, one plausible explanation is that filbertone acts, at least in part, upstream of p53 by limiting oxidative stress and/or DNA damage accumulation, rather than by directly inhibiting p53 itself. This possibility is consistent with previous reports that filbertone activates ROS-responsive PERK-TFEB autophagy-lysosomal signaling in neurons [16] and induces Nrf2-dependent antioxidant gene expression to attenuate neuronal damage [17], suggesting that filbertone may similarly enhance antioxidant defense and/or autophagic clearance of damaged organelles (e.g., mitochondria, via mitophagy) in hepatocytes, thereby reducing the DNA damage-response signals that converge on p53. Alternatively, or in addition, filbertone may act as a senomorphic rather than a senolytic agent: our data show that filbertone reduces p53–p21 signaling and suppresses the SASP in cells that remain viable (Supplemental Figure S2), which is more consistent with suppression of the senescent phenotype (senomorphic activity) than with selective clearance of senescent cells (senolysis). This distinction parallels the behavior of several well-characterized natural senotherapeutics: at low micromolar concentrations, resveratrol acts primarily as a senomorphic/antioxidant compound that suppresses the SASP without eliminating senescent cells, the combination of dasatinib and quercetin is a prototypical senolytic that selectively induces apoptosis of senescent cells, and curcumin has also been reported to modulate senescence-associated inflammatory signaling in a largely senomorphic manner [40]. The present findings position filbertone within this senomorphic category of dietary bioactive compounds, although direct head-to-head comparisons with these reference compounds, as well as mechanistic studies employing pharmacological or genetic inhibition of ROS, DNA damage-response kinases (e.g., ATM/ATR), or Nrf2, will be required to formally establish causality and to determine whether p53 is mechanistically required for filbertone’s anti-senescent effects. Direct functional validation of these hypotheses—for example, using mitochondrial ROS probes, mitophagy flux assays, or hepatocyte-specific functional readouts such as albumin secretion or lipid handling—was beyond the scope of the present study and represents an important direction for future work.

Therefore, our observations suggest that filbertone treatment influences senescence signals associated with the p53–p21 pathways in the liver. However, additional studies are needed to further explore the precise mechanisms by which filbertone regulates these pathways. These conclusions should also be considered within the context of the experimental model used: all experiments were performed in a single immortalized hepatocyte cell line (AML12) under pharmacologically induced senescence, and no in vivo model was employed to confirm these effects or their relevance to hepatic aging. Likewise, while filbertone alone was non-cytotoxic across the concentrations tested (Supplemental Figure S2), cell viability was not separately verified under the combined doxorubicin/H_2_O_2_-plus-filbertone conditions used in Figure 2 and Figure 5, and future work should include such combined viability controls to further exclude confounding cytotoxic effects. Furthermore, the filbertone concentrations used here (25–100 μM) were chosen based on prior in vitro work in other cell types [22]; because pharmacokinetic and bioavailability data establishing whether comparable hepatic concentrations are physiologically achievable through dietary hazelnut intake are not yet available [18], the present results are best regarded as evidence of a pharmacological, mechanistic effect rather than a demonstrated dietary benefit. In conclusion, filbertone attenuates p53–p21-associated senescence signaling and the accompanying SASP response in AML12 hepatocytes, supporting its further investigation as a candidate senomorphic, dietary bioactive compound for hepatic senescence-targeted functional food development, pending validation in physiologically relevant in vivo models.

## 5. Conclusions

In this study, filbertone dose-dependently attenuated doxorubicin- and H2O2-induced p53 and p21 expression, reduced SA-β-gal-positive senescent cell numbers, and suppressed the SASP-associated pro-inflammatory cytokines Il-1β, Il-6, and Tnf-α in AML12 hepatocytes, in both acutely stress-induced and established senescent-cell models. These findings indicate that filbertone modulates senescence-associated p53–p21 signaling and the accompanying inflammatory secretory phenotype in this in vitro hepatocyte model. Given the limitations of the single-cell-line, in vitro design discussed above—including the absence of in vivo validation and of direct evidence that p53 is mechanistically required for these effects—filbertone should be regarded as a candidate senomorphic, dietary bioactive compound for hepatic senescence-targeted functional food development, warranting further mechanistic and in vivo studies before any conclusions regarding a physiological anti-aging benefit can be drawn.

## Data Availability

The raw data supporting the conclusions of this article will be made available by the authors on request.

## Supplementary Materials

The following supporting information can be downloaded at: https://www.mdpi.com/article/10.3390/nu18142278/s1, Figure S1: Dose-dependent induction of p53 and p-p53 by doxorubicin in AML12 hepatocytes (6 h and 24 h); Figure S2: Effects of filbertone on the viability of AML12 hepatocytes (MTT assay); Figure S3: Time-dependent induction of p53 and p-p53 by hydrogen peroxide in AML12 hepatocytes (15–60 min); Table S1: qRT-PCR primer sequences used in this study.
