# Supplementary material for: Hepatic Cellular Senescence Is Attenuated by Filbertone via Modulation of the p53-p21 Pathway in AML12 Hepatocytes
Source: Nutrients. 2026 Jul 11;18(14):2278. doi: 10.3390/nu18142278 (PMC13414691; doi:10.3390/nu18142278)
Supplement: Supplementary file 1 [file nutrients-18-02278-s001.zip › nutrients-4354794-supplementary.pdf]

Supplemental Figure S1.

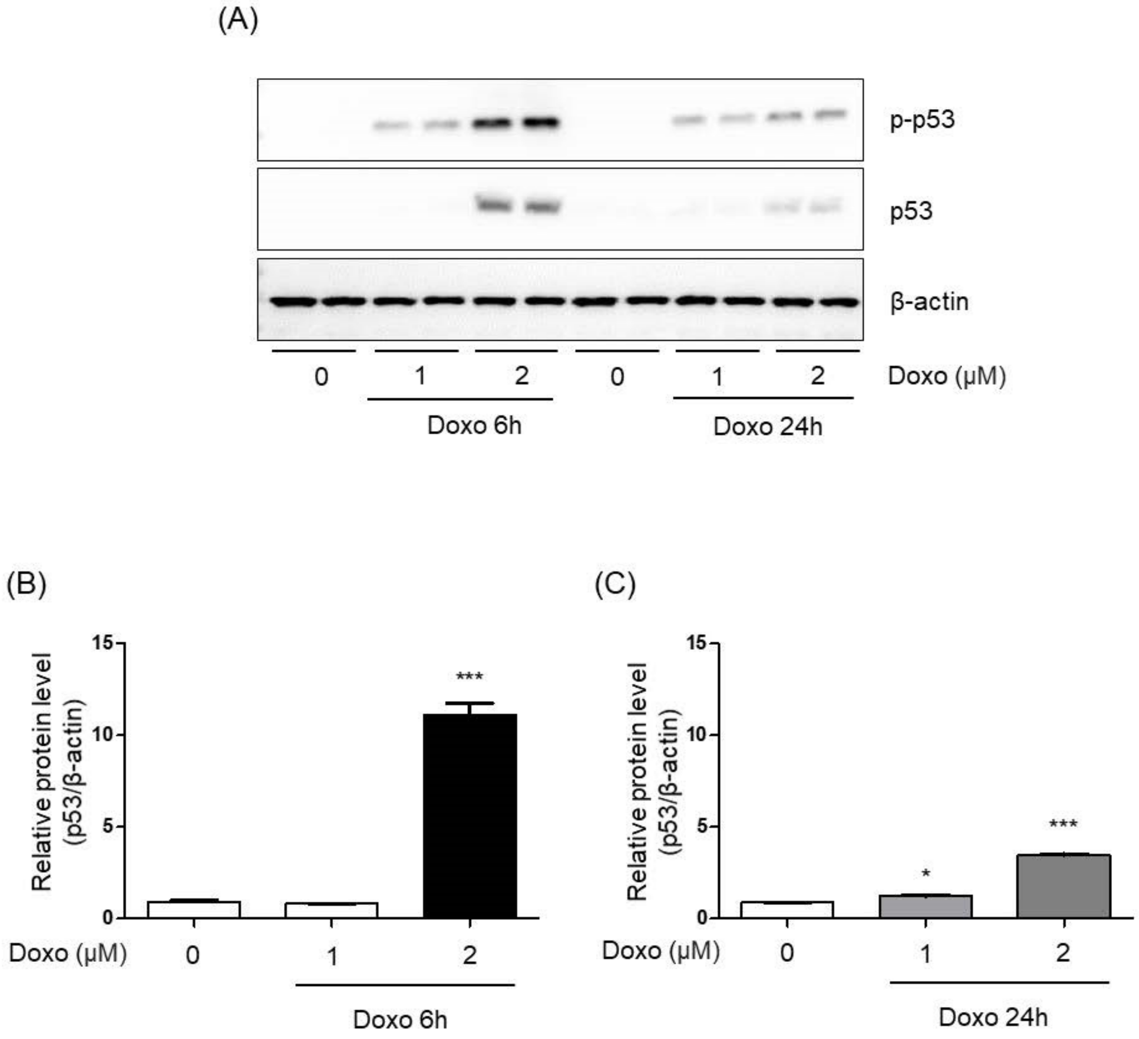

Supplemental Figure S1. Induction of cellular senescence with doxorubicin in AML12 hepatocytes.

(A) The effect of doxorubicin (Dox; 1 or 2 μM) was tested in AML12 hepatocytes for the indicated times. The protein expression of p53, p-p53, and β-actin were determined by western blot analysis. Protein levels were normalized to β-actin levels in each sample (B and C). The graph shows the quantification of Western blot data (n=4 per group). The results are expressed as mean±SD. * $p<0.05$, ** $p<0.01$, *** $p<0.005$ compared to 0h by one-way ANOVA with Tukey multiple comparison post-hoc test.

Supplemental Figure S2.

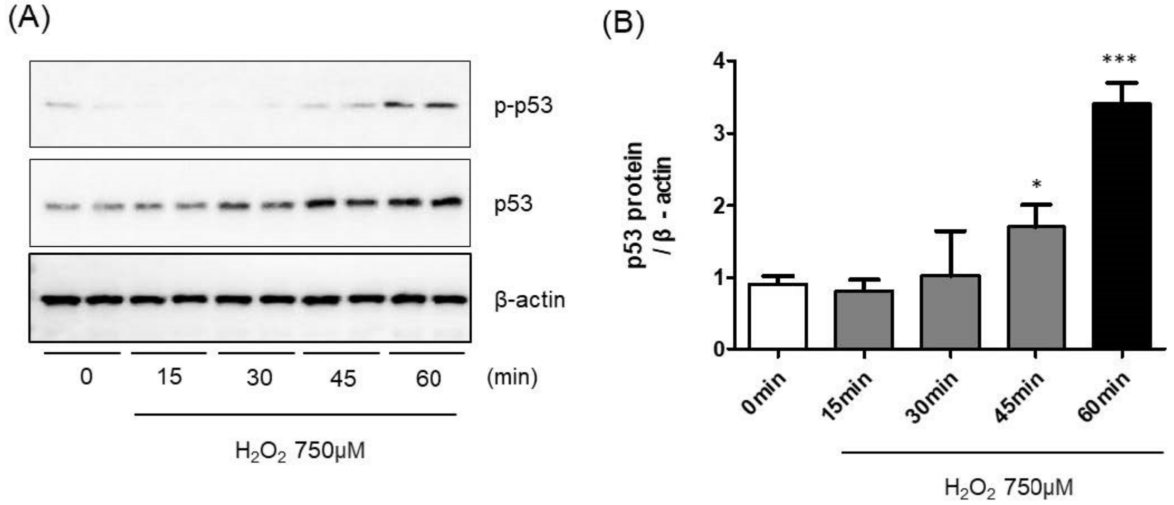

Supplemental Figure S2. Induction of cellular senescence with H_2O_2 in AML12 hepatocytes.

(A) The effect of H_2O_2 (750μM) was tested in AML12 hepatocytes for 15, 30, 45, and 60 minutes. The protein expression of p53, p-p53, and β-actin were determined by western blot analysis. Protein levels were normalized to β-actin levels in each sample (B and C). The graph shows the quantification of Western blot data (n=4 per group). The results are expressed as mean±SD. * $p<0.05$, ** $p<0.01$, *** $p<0.005$ compared to 0min by one-way ANOVA with Tukey multiple comparison post-hoc test.

Supplemental Figure S3.

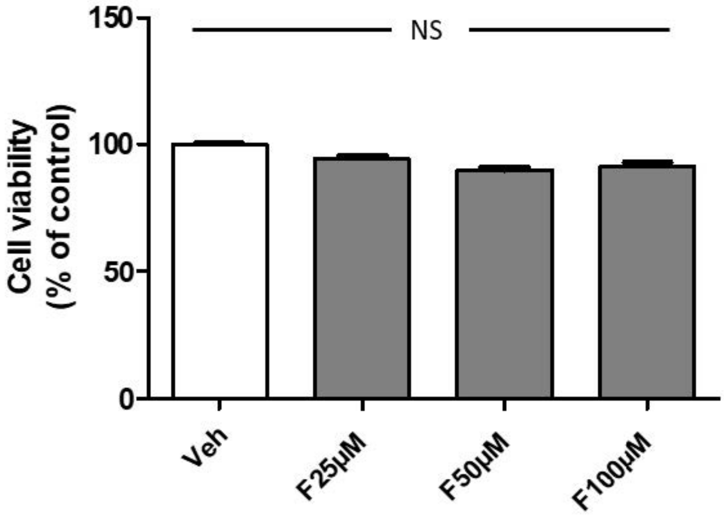

Supplemental Figure S3. Effects of filbertone on the viability of AML12 hepatocytes.

After treating AML12 cells with filbertone at concentrations of 0, 25, 50, and 100μM for 48 hours, cell toxicity was assessed through the MTT assay (n=4 per group). The cell viability is expressed as % of cell viability in comparison to a control (Veh). The data represent mean±SD. NS (not significant) is compared with Veh by one-way ANOVA with Tukey's multiple-comparisons post hoc test.

Supplemental Table S1. Mouse primer sequence for qRT-PCR

Mouse Gene	Forward (5' to 3')	Reverse (5' to 3')
Trp53	CTCCGAAGACTGGATGACTG	ACAGATCGTCCATGCAGTGAG
Cdkn1a (p21)	TGTCTTGCACTCTGGTGTCTG	CAATCTGCGCTTGGAGTGAT
Rplp0 (36B4)	TGGAAGTCCAACTACTTCCTCAA	ATCTGCTGCATCTGCTTGGAG
